# On the Therapeutic Potential of Heme Oxygenase-1 and Its Metabolites

**DOI:** 10.3390/antiox13101243

**Published:** 2024-10-16

**Authors:** David E. Stec

**Affiliations:** Cardiorenal and Metabolic Diseases Research Center, Department of Physiology and Biophysics, University of Mississippi Medical Center, Jackson, MS 39216, USA; dstec@umc.edu

Over the past 55 years, the heme oxygenase (HO) system has emerged as a pivotal player in a myriad of cellular, tissue, and integrative physiological processes [1,2]. Heme, a crucial component of various proteins, including cytochrome P450 enzymes, hemoglobin, myoglobin, and mitochondrial respiratory proteins, releases free heme upon degradation, necessitating recycling by HO enzymes [3]. However, increased cell-free heme levels resulting from hemolysis are associated with chronic widespread pain (CWP), especially in patients with HIV infection [4]. Carbon monoxide (CO) gas and biliverdin are generated as part of the breakdown and recycling of heme. Biliverdin is then reduced to bilirubin by the enzyme biliverdin reductase.

The HO pathway serves a complex physiological function that, under some circumstances, is beneficial; in others, it is deleterious, highlighting its dual role. For example, in cancer, HO promotes certain types of cancers (head and neck) and can limit the effectiveness of chemotherapeutic agents [5,6]. In this case, the inhibition or genetic knockdown of HO could improve the effectiveness of chemotherapeutic agents. However, certain anti-cancer agents can induce HO-1 to aid in treating certain types of cancers. Hyperforin, for example, is an extract of St. John’s wort that triggers melanoma cell apoptosis and represses the expression of pro-metastatic markers in an HO-1-dependent fashion [7]. More work needs to be carried out to completely understand the complex role of HO-1 in cancer biology and maximize effective therapeutics targeting it.

HO-1 plays an important role in regulating obesity and diabetes [8]. The induction of HO-1 in adipocytes results in decreased fat mass, improved insulin resistance, and decreased levels of adipose-derived cytokines [9]. Peroxisome proliferator-activated receptor gamma coactivator 1-alpha (PGC-1α) is a transcription factor that regulates genes involved in metabolism. Adipocyte-specific overexpression of PGC-1α browns white adipocytes and improves insulin resistance in an HO-1-dependent fashion [10]. The effects of HO-1 induction in obesity are likely due to the increased production of CO and bilirubin, as both of these products can lower body weight and improve insulin resistance [11,12].

The bilirubin/biliverdin reductase pathway is an emerging area of research for several different diseases. Biliverdin reductase A (BVRA) has been shown to play an important role in metabolic diseases like diabetes, nonalcoholic fatty liver disease, and Alzheimer’s disease [13,14,15,16]. Therefore, the development of agents to induce BVRA could be beneficial for these diseases. Moon et al. demonstrated that Korean red ginseng extract (KRGE) induces BVRA expression, which plays a critical role in its protective effects on the central nervous system [17]. The BVRA product, bilirubin, is important in cardiovascular and metabolic diseases. Kipp et al. demonstrated that bilirubin levels are negatively correlated with adiposity, while the levels of the bilirubin breakdown product, urobilin, are positively associated with insulin resistance. [18]. These results indicate the opposite effects of bilirubin and its breakdown products on metabolism. They also indicate that therapies that alter the bilirubin–urobilin ratio could be effective against obesity and diabetes. Low serum bilirubin levels are associated with atherosclerosis and increased proinflammatory markers [19].

HO is also a critical inflammatory compound. Its induction by numerous compounds, including kaempferol, a flavonoid found in various plants, attenuates inflammation via several pathways. For example, in human pulmonary alveolar epithelial cells, kaempferol attenuates intracellular cell adhesion protein 1 (ICAM-1) expression via an HO-dependent pathway [20]. Similarly, the induction of HO-1 via 15-Deoxy-Δ [12,14] prostaglandin J_2_ (15d-PGJ_2_) suppresses lipopolysaccharide-induced inflammation in endothelial cells [21]. Thus, developing HO inducers could be a key component for anti-inflammatory diseases.

The induction of HO-1 provides beneficial effects in numerous models of cardiovascular disease [22]. Abdominal aortic aneurysm (AAA) is a vascular pathology characterized by the accumulation of inflammatory cells, increased local cytokine production, oxidative stress, and ultimately the loss of medial vascular smooth muscle cells. One of the main treatment approaches for AAA is regulating blood pressure through several anti-hypertensives, including renin-angiotensin system blockers, calcium channel blockers, and diuretics. Hofmann et al. demonstrated that several of these therapeutic approaches were associated with the induction of HO-1 in aortic tissue, suggesting an essential role for HO-1 in the vascular protection offered by these treatments [23].

While there is ample evidence for the development of HO-based therapeutics, the process of developing these drugs has yet to be fully explored. In a ground-breaking study, Ghajar-Rahimi et al. used a novel cell-based, high-throughput screen for inducing HO-1 via the human HO-1 promoter/enhancer driving luciferase expression [7]. Through this method, they identified two new small-molecule inducers of HO-1 (SRI-37618 and SRI-40109) and the antiprotozoal agent broxaldine as novel HO-1 inducers [24]. This work demonstrates how novel HO-1 inducers can potentially be developed on a large scale to treat several cardiovascular, renal, and metabolic diseases.

This Special Issue has highlighted the important role of HO, its associated protein BVRA, and bilirubin in several important research areas. It reflects the HO system’s diverse roles in many diseases and highlights the most innovative research. Through progress in our understanding of the HO system, novel therapeutics targeting this pathway can be developed in the future to combat several pathological diseases. We want to extend our deepest appreciation to all of the authors who contributed their work to this Special Issue.
